# Dangerous sweets: severe hypokalemia with rhabdomyolysis and tetraparesis from chronic consumption of licorice

**DOI:** 10.1007/s00415-020-10347-y

**Published:** 2020-12-19

**Authors:** Matthias Wittstock, Allessandro Mele, Daniel Cantré, Alexander Storch

**Affiliations:** 1 Department of Neurology, University Medicine Centre Rostock, University of Rockstock, Gehlsheimer Str. 20, 18147 Rostock, Germany; 2 Department of Diagnostic and Interventional Radiology, University Medical Centre Rostock, University of Rockstock, Rockstock, Germany; 3 Department of Pediatric Radiology and Neuroradiology, University Medical Centre Rostock, University of Rockstock, Rockstock, Germany

Dear Sirs,

Recently, Edelman and colleagues^1^ reported the case of a 44-year-old man with pseudohyperaldosteronism suggestive of excessive licorice consumption complicated by cardiac arrest associated with ventricular fibrillation. Beside these severe cardiac complications, acute non-traumatic tetraparesis may be observed as a symptom of severe hypokalemia due to liquorice consumption.

A 67-year-old female was admitted with an acute, non-traumatic, painless tetraparesis. Neurological exam revealed slight dysarthria, but no facial or bulbar weakness, a moderate tetraparesis (MRC level 3 in the lower limbs and MRC level 4 in the upper limbs) with only slightly weakened deep tendon reflexes. No sensory level could be shown. Upon admission, there was nausea with vomiting, a pathologically increased blood glucose level and bradycardia. Cranio-cervical computed tomography showed normal findings. The ECG did not show a prominent U wave. The admission laboratory showed severe hypokalemia (1.7 mmol/l) and rhabdomyolysis with a serum creatine kinase of 2372 U/l (**Table ****1**). No causes for an infectious or drug-induced hypokalemia could be determined. Nephropathy and a mass of the adrenal cortex were excluded by ultrasound. There was no laboratory evidence of metabolic-endocrine disorders such as primary and secondary hyperaldosteronism or hypercortisolism. We observed pathologically low levels of aldosterone (67 pmol/l, normal range: 69–634 pmol/liter) and renin (1.5 pg/ml, normal range: 3–22) with borderline elevated cortisol levels (601 nmol/liter, normal range: 133–537). Further exploration of the patient revealed a daily consumption of unusually high amounts of sweets containing liquorice (approx. 250 g, corresponding to approx. 7.5 mg of contained liquorice). A regression of the paresis was already evident 72 h after parenteral potassium substitution. The delayed increase of CK levels underlines the most probable mechanism of liquorice caused hypokalemia inducing vasoconstriction with relative ischemia in the active muscles leading to subsequent necrosis and rhabdomyolysis. The patient could be discharged without any neurological sequelae.

**Table 1:** Laboratory data

Laboratory value	Reference range, adults	Hospital admission 25.04.2020 (13:30)	25.04.2020 (19:30)	26.04.2020 (8:30)	27.04.2020 (7:15)	27.04.2020 (14:30)	Hospital discharge on 29.04.2020 (7:30)
Blood							
Sodium (mmol/liter)	136–144	137	138		145	141	
Potassium (mmol/liter)	3.6–5.1	1.7	1.6		3.4	4.0	
Chloride (mmol/liter)	98–107	99				111	
Calcium (mmol/l)	2.20–2.65	2.24			2.00	1.94	
Creatine kinase (U/l)	< 170	2,372	1,742	1,439	6,411	7,750	5,327
Myoglobine (ng/ml)	25–58				2911		711
Hormones							
TSH (µU/ml)	0.27–4.2	0.521					
fT3 (pmol/l)	3.13–6.76	4.55					
fT4 (pmol/l)	11.6–24.6	21.1					
Aldosterone basal level (pmol/l)	69–634						67
Renine (pg/ml)	3–22						1.5
Cortisol (nmol/l)	133–537						601

The substance glycyrrhizin contained in licorice is an inhibitor of the enzyme 11β-hydroxysteroid dehydrogenase being responsible for inactivating cortisol by converting it into cortisone. The total amount of glycyrrhizin is very variable in the different liquorice mixtures. The exact content in our patient´s mixture was not declared. Due to the mineralocorticoid effect of cortisol on the aldosterone receptors, our patient shows the typical laboratory constellation of pseudohyperaldosteronism.^2^ This hypokalemic metabolic condition may be differentiated from Liddle, Bartter or Gitelman syndrome by blood pressure, age of onset, biochemistry***** and negative family history.^3^ Rhabdomyelysis or neurological deficits have been reported occasionally.^4^ Remarkably, already relatively small amounts of liquorice in our patient compared with reported cases (< 10 mg vs. 300 g daily)^5^ may lead to severe consecutive neurological deficits. This case illustrates the range of possible cardiologic and neurological problems due to hypokalemia and emphasizes the importance of a detailed anamnesis with a special focus on the eating habits of patients showing dyselectrolytemia and neurological deficits.

## References

[1] Edelmann ER, Butala NM, Avery LL, Lundquist AL, Dighe AS (2020). Case 30–2020: A 54-year-old man with sudden cardiac arrest. New Engl J Med.

[2] Kato H, Kanaoka M, Yano S, Kobayashi M (1995). 3-Monoglucuronyl-glycerhetinic acid is a major metabolite that causes licorice-induced pseudoaldosteronism. J Clin Endocrinol Metab.

[3] Munford E, Unwin RJ, Walsh SB (2019). Liquorice, Liddle, Bartter or Gitelmann – how to differentiate?. Nephrol Dial Transplant.

[4] Rong HE, Guo WJ, She F, Miao GB, Liu F, Xue YJ, Liu YW, Wang HT, Zhang P (2018). A rare case of hypokalemia-induced rhabdomyolyis. J Geriatr Cardiol.

[5] Attout H, Randriajohany A, Josse F, Appavoupoule V, Thirapathi Y. Tetraparesis with major hypokalaemia and rhabdomyolysis induced by chronic liquorice ingestion. Eur J of Case Reports in Internal Med 2020; 7; DOI: 10.12890/2020_001375. 10.12890/2020_001375 PMC7162572 32309249

